# Globally Optimal Distributed Fusion Filter for Descriptor Systems with Time-Correlated Measurement Noises

**DOI:** 10.3390/s22197469

**Published:** 2022-10-02

**Authors:** Jing Ma, Liling Xu

**Affiliations:** School of Mathematics Science, Heilongjiang University, Harbin 150080, China

**Keywords:** distributed fusion, filter, descriptor system, time-correlated noise, global optimality

## Abstract

This paper concerns the distributed fusion filtering problem for descriptor systems with time-correlated measurement noises. The original descriptor is transformed into two reduced-order subsystems (ROSs) based on singular value decomposition. For the first ROS, a new measurement is obtained using measurement difference technology. Each sensor produces a local filter based on the fusion predictor from the fusion center and its own new measurement and then sends it to the fusion center. In the fusion center, based on local filters, a distributed fusion filter with feedback (DFFWF) in the linear minimum variance (LMV) sense is proposed by applying an innovative approach. The DFFWF for the second ROS is also obtained based on the DFFWF for the first ROS. Then, the DFFWF for the original descriptor is obtained. The proposed DFFWF can achieve the same estimation accuracy as the centralized fusion filter (CFF) under the condition that all local filter gain matrices are of full column rank. Its optimality is strictly proved. Moreover, it has robustness and reliability due to the parallel processing of local filters. Two simulation examples demonstrate the effectiveness of the developed fusion algorithm.

## 1. Introduction

In the last few decades, the problem of state estimation for descriptor (singular) systems has attracted much attention due to more widespread applications than normal systems, such as in power systems, electrical networks, chemical processes, social–economic systems, network analysis, constrained mechanical systems, time-series analysis, large-scale systems with interconnections, aerospace attitude control systems and so on [1,2,3].

Generally speaking, there are two common methods that deal with the filtering problem for descriptor systems: the full-order transformation method and the reduced-order decomposition method. Based on the full-order transformation method, the optimal linear estimators for single sensor systems [4] and distributed fusion estimators for multi-sensor systems [5] are proposed, which are directly solved based on the projection theory. However, the derivation of the estimator is complex since the descriptor system with white noise is transformed into a normal system with one-step cross-correlation colored noise. Differently from full-order methods [4,5], the original descriptor system is equivalently decomposed into two reduced-order subsystems based on the singular value decomposition, where the first reduced-order system is the normal system with white noise. Hence, the reduced-order decomposition method is more popular. Based on the reduced-order decomposition method, many estimators have been reported, including the linear minimum mean-square filter for a single sensor system with stochastic multiplicative disturbance [6], the distributed weighted state fusion optimal filter [7] and steady-state estimators [8] for systems with correlated white noise, distributed weighted state fusion filter for a system with fading measurements and stochastic nonlinearity [9], weighted measurement fusion robust estimators [10] and self-tuning estimators [11], and centralized fusion estimators for systems with different delay rates [12]. However, the above works do not consider the time-correlated measurement noises.

In recent years, research on time-correlated noises has gained lots of attention because of its extensive application background in practical areas such as the modern radar systems [13], the global navigation satellite systems and the unreliable network communication systems [14], etc [15,16]. Up to now, there are two common methods to deal with the time-correlated noises: the state augmented method and the measurement difference method [17,18,19,20,21,22,23]. State augmented method is direct and simple. However, the potential ill-conditioned problem and expensive computational burden are the main shortcomings [17,18]. In comparison, the measurement difference method has more advantages. It can avoid the ill-conditioned problem of the estimation error covariance matrix and reduce the computational cost. The measurement difference methods are also divided into forward difference and backward difference. The future measurements are required to update the filter in forward measurement difference, which leads to a delay in the output of the filter [19]. Hence, the most popular method is the backward measurement difference method. Based on it, many benefit results have been proposed such as linear least variance filters [18,20], a modified Tobit Kalman filter [21], the innovation-based and consensus-based distributed fusion filters by considering neighboring filters [22], distributed and centralized fusion filter and smoother [23], optimal linear filter for singular system [24].

In recent years, information fusion state estimation problem has become the hot topic in signal processing, target tracking, navigation and positioning areas [25]. The common methods are centralized and distributed fusion and sequential fusion. For asynchronous or delayed data, sequential fusion is more convenient [26]. When all sensors are healthy, centralized fusion can give the globally optimal estimation results in the LMV sense. Distributed fusion is easy to detect and isolate the faulty sensors since the local filters are processed in parallel. So far, many distributed fusion estimators for normal system with time-uncorrelated white noises have been reported including the weighted state fusion filter in LMV sense [27], fusion filter based on information filter [28], and suboptimal and globally optimal distributed fusion filters without/with feedback [29,30,31]. Under some conditions, the above distributed optimal fusion filters [28,29,30,31] can achieve the globally optimal estimation accuracy in LMV sense. In the recent studies [32,33,34], some new improved distributed fusion strategies have been proposed. For nonlinear integrated unmanned aerial vehicle navigation system, a new cubature rule-based distributed fusion strategy has been proposed in [32]. The developed fusion technique can effectively identify and predict kinematic model error and achieve globally optimal fusion results. In [33], a novel low-complexity reduced-order fusion filter is designed by fusing a subset of state components rather than all state variables. In [34], based on reduced dimension hypercomplex technique, the centralized and distributed prediction and smoothing fusion algorithms for system with uncertain measurements are proposed in the tessarine domain. However, to the best of the author’s knowledge, the globally optimal distributed fusion filter for descriptor system with time-correlated measurements has not been reported.

Motivated by the above analysis, the state estimation problem for systems with time-correlated noises has not been fully solved. Most of the existing works are the linear filter for normal system measured by single sensor. In the current paper, we focus on the DFFWF for descriptor systems with time-correlated measurement noise. Based on singular value decomposition method and backward measurement difference method, a DFFWF is presented in the LMV sense. The developed DFFWF can achieve the same accuracy as the centralized fusion one. The optimality of the DFFWF is rigorously proved. Moreover, the proposed DFFWF avoids the complex computation of the cross-covariance matrices between any two local estimation errors in the distributed weighted fusion method.

Notation: ${ℜ}^{n}$ is the n dimensional Euclidean space. diag(•) represents the diagonal matrix; 0 represents the zero matrix with the suitable dimensions; $I_{m_{k}}$ represents $m_{k}\times m_{k}$ identity matrix. $A^{+}$ signifies the Moore-Penrose inverse of a matrix A. $\delta_{t,k}$ is Kronecker delta function. Superscript Τ denotes transpose of a matrix. Ε[•] is the mathematical expectation operator. proj{•} denotes the projection operator. x⊥y means random variables x and y are uncorrelated, i.e., $Ε[xy^{T}]=0$. $L(y_{j}(1),\cdots ,y_{j}(t-1),y_{j}(t))$ stands for the linear space spanned by the measurement sequence $\left\{y_{j}(1),\cdots ,y_{j}(t-1),y_{j}(t)\right\}$. Subscript j denotes the *j*th sensors, N denotes the number of the sensors.

## 2. Problem Formulation and Preliminary Lemmas

Consider the following multi-sensor stochastic descriptor system with time-correlated measurement noises:

(1) $$\bar{A}x(t+1)=\bar{B}x(t)+\bar{C}w(t),$$

(2) $$y_{j}(t)={\bar{D}}_{j}x(t)+v_{j}(t),\;j=1,\ldots ,N,$$

(3) $$v_{j}(t+1)={\bar{U}}_{j}v_{j}(t)+\mu_{j}(t),$$

where $x(t)\in {ℜ}^{n}$ is the state and $y_{j}(t)\in {ℜ}^{m_{j}}$ is the measurement output. $\bar{A},\bar{B},\bar{C},{\bar{D}}_{j},{\bar{U}}_{j}$ are known constant parameter matrices with proper dimensions..

We make the following assumptions.

**Assumption** **1.** $\bar{A}$*is a singular square matrix, i.e.,* $rank(\bar{A})=n_{1}<n$.

**Assumption** **2.** *Systems (1)–(3) are regular,* i.e.,$\det(s\bar{A}-\bar{B})\neq 0$ *, and* s *is an arbitrary complex*.

**Assumption** **3.** $rank(\bar{B})=n$.

**Assumption** **4.** w(t) and $\mu_{j}(t)$ are uncorrelated white noises with zero means and covariance matrices $E[w(t)w^{T}(k)]=Q^{w}\delta_{t,k}$ and $E[\mu_{j}(t)\mu_{j}^{T}(k)]=Q_{j}^{\mu }\delta_{t,k}$,j=1,…,N.

**Assumption** **5.** *The initial state value* x(0)*and measurement noise initial values*$v_{j}(0)$*,*j=1,⋯,N*aremutually uncorrelated and are independent of*w(t)*and*$\mu_{j}(t)$*, and satisfy*$E[x(0)]=x_{0}$*,*$E[(x(0)-x_{0}){\left(x(0)-x_{0}\right)}^{T}]=P_{0}$*,*$E[v_{j}(0)]=v$*,*$E[(v_{j}(0)-v_{j}){\left(v_{j}(0)-v_{j}\right)}^{T}]=P_{0}^{v_{j}}$.

Our aim is to design the globally optimal DFFWF ${\hat{x}}_{df}(t|t)$ in the LMV sense. Besides, the global optimality of the DFFWF is proved.

**Remark** **1.** *Descriptor systems appear in many fields, such as electrical circuit systems, large-scale systems with interconnections, constrained mechanical systems. Some concrete examples of descriptor systems are presented in [1], from which readers can indeed see the existence of descriptor linear systems in our real world. In the simulation research section, an electrical circuit system is used to show the effectiveness of the proposed DFFWF algorithm.*

### System Transformation

Under Assumption 3, there exist the non-singular matrices $\bar{M}$ and $\bar{N}$ [7,8], which satisfy

(4) $$\bar{M}\bar{A}\bar{N}=\left[\begin{array}{cc} {\bar{A}}_{1} & 0\\ {\bar{A}}_{2} & 0 \end{array}\right],\;\bar{M}\bar{B}\bar{N}=\left[\begin{array}{cc} {\bar{B}}_{1} & 0\\ {\bar{B}}_{2} & {\bar{B}}_{3} \end{array}\right],\;{\bar{D}}_{j}\bar{N}=[{\bar{D}}_{j}^{\left(1\right)},{\bar{D}}_{j}^{\left(2\right)}],\;\bar{M}\bar{C}=\left[\begin{array}{c} {\bar{C}}_{1}\\ {\bar{C}}_{2} \end{array}\right],\;$$

where ${\bar{A}}_{1}\in {ℜ}^{n_{1}\times n_{1}}$ and ${\bar{B}}_{3}\in {ℜ}^{\left(n-n_{1})\times (n-n_{1}\right)}$ are both non-singular lower triangular matrices, ${\bar{B}}_{1}\in {ℜ}^{n_{1}\times n_{1}}$ is the quasi-lower triangular matrix and ${\bar{A}}_{2}$, ${\bar{B}}_{2}$, ${\bar{C}}_{1}$, ${\bar{C}}_{2}$, ${\bar{D}}_{j}^{\left(1\right)}$, ${\bar{D}}_{j}^{\left(2\right)}$, j=1,…,N are matrices with appropriate dimensions. By introducing $x(t)=\bar{N}{\left[\begin{array}{cc} {\left(x^{\left(1\right)}(t)\right)}^{T}, & {\left(x^{\left(2\right)}(t)\right)}^{T} \end{array}\right]}^{T}$, the original descriptor system can be transformed into the following two ROSs:

(5) $$\left\{ \begin{array}{l} x^{\left(1\right)}(t+1)=\Phi x^{\left(1\right)}(t)+\Gamma w(t),\\ y_{j}(t)={\bar{H}}_{j}x^{\left(1\right)}(t)+{\bar{D}}_{j}^{\left(2\right)}Cw(t)+v_{j}(t)\\ v_{j}(t+1)={\bar{U}}_{j}v_{j}(t)+\mu_{j}(t), \end{array}\right.,$$

(6) $$x^{\left(2\right)}(t)=Bx^{\left(1\right)}(t)+Cw(t),\;$$

where $\Phi ={\bar{A}}_{1}^{-1}{\bar{B}}_{1}$, $\Gamma ={\bar{A}}_{1}^{-1}{\bar{C}}_{1}$, ${\bar{H}}_{j}={\bar{D}}_{j}{}^{\left(1\right)}+{\bar{D}}_{j}{}^{\left(2\right)}B$, $B={\bar{B}}_{3}^{-1}{\bar{A}}_{2}{\bar{A}}_{1}^{-1}{\bar{B}}_{1}-{\bar{B}}_{3}^{-1}{\bar{B}}_{2}$ and $C={\bar{B}}_{3}^{-1}{\bar{A}}_{2}{\bar{A}}_{1}^{-1}{\bar{C}}_{1}-{\bar{B}}_{3}^{-1}{\bar{C}}_{2}$.

It is clear that the first ROS (5) is a normal system with time-correlated measurement noise $v_{j}(t)$, and the second ROS (6) is a linear combination of $x^{\left(1\right)}(t)$ and w(t).

First, we adopt the measurement difference method used in ref. [20] to remove the time-correlated noise $v_{j}(t)$. Using the measurement difference, the new measurement can be expressed as:

(7) $$z_{j}(t)=y_{j}(t)-{\bar{U}}_{j}y_{j}(t-1)=({\bar{H}}_{j}-{\bar{U}}_{j}{\bar{H}}_{j}\Phi^{-1})x^{\left(1\right)}(t)+{\bar{D}}_{j}^{\left(2\right)}Cw(t)\;+{\bar{U}}_{j}({\bar{H}}_{j}\Phi^{-1}\Gamma -{\bar{D}}_{j}^{\left(2\right)}C)w(t-1)+\mu_{j}(t-1).$$

In the above derivation, we use the fact that $x^{\left(1\right)}(t-1)=\Phi^{-1}(x^{\left(1\right)}(t)-\Gamma w(t-1))$ according to the state update equation in Equation (5). This is acceptable since the state transition matrix must be invertible [20,35].

Then, the first ROS can be expressed as:

(8) $$\left\{ \begin{array}{l} x^{\left(1\right)}(t+1)=\Phi x^{\left(1\right)}(t)+\Gamma w(t),\\ z_{j}(t)=D_{j}x^{\left(1\right)}(t)+\eta_{j}(t),\\ \eta_{j}(t)=J_{j}w(t)+F_{j}w(t-1)+\mu_{j}(t-1), \end{array}\right.$$

where $D_{j}={\bar{H}}_{j}-{\bar{U}}_{j}{\bar{H}}_{j}\Phi^{-1}$, $J_{j}={\bar{D}}_{j}^{\left(2\right)}C$ and $F_{j}={\bar{U}}_{j}({\bar{H}}_{j}\Phi^{-1}\Gamma -{\bar{D}}_{j}^{\left(2\right)}C)$.

**Remark** **2.** *It is clear from Equation (8) that the new measurement noise* $\eta_{j}(t)$*is a one-step auto-correlationand cross-correlation with process noise* w(t)*, which brings achallengeto obtaining the globally optimal linear filter.*

For the sake of convenience in discussion, we introduce the augmented vectors: $z(t)={\left[z_{1}^{T}(t),\cdots ,z_{N}^{T}(t)\right]}^{T}$, $\eta (t)={\left[\eta_{1}^{T}(t),\cdots ,\eta_{N}^{T}(t)\right]}^{T}$, $\mu (t)={\left[\mu_{1}^{T}(t),\cdots ,\mu_{N}^{T}(t)\right]}^{T}$, $D={\left[D_{1}^{T},\cdots ,D_{N}^{T}\right]}^{T}$, $F={\left[F_{1}^{T},\cdots ,F_{N}^{T}\right]}^{T}$ and $J={\left[J_{1}^{T},\cdots ,J_{N}^{T}\right]}^{T}$. Then, the augmented system can be written as:

(9) $$\left\{ \begin{array}{l} x^{\left(1\right)}(t+1)=\Phi x^{\left(1\right)}(t)+\Gamma w(t),\\ z(t)=Dx^{\left(1\right)}(t)+\eta (t),\\ \eta (t)=Jw(t)+Fw(t-1)+\mu (t-1). \end{array}\right.$$

Further, we determined the following noise statistic information using Assumptions 4 and 5, which play an important role in the design of DFFWF.

(10) $$E[\mu (t)\mu^{T}(t)]=Q^{\mu },\;E[\eta (t)\eta^{T}(t-k)]=Q^{\eta }(k),\;E[w(t)\eta^{T}(t-l)]=Q^{w\eta }(l),\;\;Q^{\mu }=\mathrm{diag}(Q_{1}^{\mu },\cdots ,Q_{N}^{\mu }),\;Q^{\eta }(0)=JQ^{w}J^{T}+FQ^{w}F^{T}+Q^{\mu },Q^{\eta }(1)=FQ^{w}J^{T},\;\;Q^{\eta }(-1)=JQ^{w}F^{T},\;Q^{w\eta }(0)=Q^{w}J^{T},\;Q^{w\eta }(-1)=Q^{w}F^{T}.\;k=0,\pm 1,\;l=0,-1,\;$$

**Remark** **3.** *According to Equation (9), we determine that* $x^{\left(1\right)}(t)\in L(w(t-1)\cdots ,w(0),x^{\left(1\right)}(0))$*and* $L(z(1),\cdots ,z(t))\subset L(w(t),w(t-1)\cdots ,w(0),x^{\left(1\right)}(0),\mu (t-1),\cdots ,\mu (0))$*, which implies* $w(t)⊥x^{\left(1\right)}(t)$, $\eta (t+k)⊥x^{\left(1\right)}(t)$, k>0 and w(t+1)⊥L(z(1),⋯,z(t)), η(t+2)⊥L(z(1),⋯,z(t)).

Before ending this section, we recall the following CFF for the considered descriptor system, which serves in the subsequent sections.

**Lemma** **1.** *For the first ROS (9) under Assumptions 1* *–5, the CFF is computed by:*

(11) $${\hat{x}}_{c}^{\left(1\right)}(t|t)=(I_{n_{1}}-K_{c}(t)D){\hat{x}}_{c}^{\left(1\right)}(t|t-1)-K_{c}(t){\hat{\eta }}_{c}^{}(t|t-1)+K_{c}(t)z(t),$$

(12) $$P_{c}^{\left(1\right)}(t|t)=P_{c}^{\left(1\right)}(t|t-1)-K_{c}(t)P_{c}^{z}(t)K_{c}^{T}(t),$$

(13) $$K_{c}(t)=(P_{c}^{\left(1\right)}(t|t-1)D_{}^{T}+P_{c}^{x\eta }(t|t-1)){\left(P_{c}^{z}(t)\right)}^{-1}.$$

*The centralized fusion predictor is computed by*

(14) $${\hat{x}}_{c}^{\left(1\right)}(t+1|t)=\Phi {\hat{x}}_{c}^{\left(1\right)}(t|t)+\Gamma {\hat{w}}_{c}^{}(t|t),$$

(15) $$P_{c}^{\left(1\right)}(t+1|t)=\Phi P_{c}^{\left(1\right)}(t|t)\Phi^{T}+\Gamma P_{c}^{w}(t|t)\Gamma^{T}-\Phi P_{c}^{xw}(t|t)\Gamma^{T}-\Gamma {\left(P_{c}^{xw}(t|t)\right)}^{T}\Phi^{T}.$$

*The new measurement noise one-step predictor is computed by*

(16) $${\hat{\eta }}_{c}(t+1|t)=-K_{c}^{\eta }(t+1|t){\hat{\eta }}_{c}^{}(t|t-1)-K_{c}^{\eta }(t+1|t)D{\hat{x}}_{c}^{\left(1\right)}(t|t-1)+K_{c}^{\eta }(t+1|t)z(t),$$

(17) $$K_{c}^{\eta }(t+1|t)=Q^{\eta }(1){\left(P_{c}^{z}(t)\right)}^{-1},$$

(18) $$P_{c}^{\eta }(t+1|t)=Q^{\eta }(0)-K_{c}^{\eta }(t+1|t)P_{c}^{z}(t){\left(K_{c}^{\eta }(t+1|t)\right)}^{T},$$

(19) $$P_{c}^{x\eta }(t+1|t)=\Gamma Q^{w\eta }(-1)-(\Phi K_{c}(t)+\Gamma K_{c}^{w}(t|t))P_{c}^{z}(t){\left(Q^{\eta }(1)\right)}^{T}.$$

*The process noise filter is computed by*

(20) $${\hat{w}}_{c}(t|t)=-K_{c}^{w}(t|t){\hat{\eta }}_{c}^{}(t|t-1)-K_{c}^{w}(t|t)D{\hat{x}}_{c}^{\left(1\right)}(t|t-1)+K_{c}^{w}(t|t)z(t),$$

(21) $$K_{c}^{w}(t|t)=Q^{w\eta }(0){\left(P_{c}^{z}(t)\right)}^{-1},$$

(22) $$P_{c}^{w}(t|t)=Q^{w}-K_{c}^{w}(t|t)P_{c}^{z}(t){\left(K_{c}^{w}(t|t)\right)}^{T},$$

(23) $$P_{c}^{xw}(t|t)=-K_{c}(t)P_{c}^{z}(t){\left(K_{c}^{w}(t|t)\right)}^{T}.$$

*with*

(24) $$P_{c}^{z}(t)=DP_{c}^{\left(1\right)}(t|t-1)D_{}^{T}+P_{c}^{\eta }(t|t-1)+DP_{c}^{x\eta }(t|t-1)+{\left(DP_{c}^{x\eta }(t|t-1)\right)}^{T},$$

*where the initial values are*${\hat{x}}_{c}^{\left(1\right)}(0|-1)=x_{0}^{\left(1\right)}$*,*${\hat{\eta }}_{c}^{}(0|-1)=0$*,*$P_{c}^{\left(1\right)}(0|-1)=P_{0}^{\left(1\right)}$*and*$P_{c}^{\eta }(0|-1)=Q^{\eta }(0)$*,*$P_{c}^{x\eta }(0|-1)=0$*, where*$x_{0}^{\left(1\right)}$*is the first*$n_{1}$*components of*${\bar{N}}^{-1}x_{0}$*, and*$P_{0}^{\left(1\right)}$*is the first*$n_{1}\times n_{1}$*sub-block of*${\bar{N}}^{-1}P_{0}{\bar{N}}^{-}{}^{T}$.

**Proof.**  The proof is similar to the case for normal systems with one-step auto- and cross-correlated measurement noises under the data receiving rate α=1 [36]. □

**Lemma** **2.** *For the second ROS (6) under Assumptions 1* *–5, the CFF is provided by:*

(25) $${\hat{x}}_{c}^{\left(2\right)}(t|t)=B{\hat{x}}_{c}^{\left(1\right)}(t|t)+C{\hat{w}}_{c}(t|t),$$

(26) $$P_{c}^{\left(2\right)}(t|t)=BP_{c}^{\left(1\right)}(t|t)B^{T}+CP_{c}^{w}(t|t)C^{T}+BP_{c}^{xw}(t|t)C^{T}+C{\left(P_{c}^{xw}(t|t)\right)}^{T}B^{T}.$$

*The cross-covariance matrix between the two subsystems is computed by*

(27) $$P_{c}^{\left(12\right)}(t|t)=P_{c}^{\left(1\right)}(t|t)B^{T}+P_{c}^{xw}(t|t)C^{T}.$$

*The fusion state filter and its filtering error covariance matrix of the original descriptor (1)–(3) are provided by*

$${\hat{x}}_{c}^{}(t|t)=\bar{N}{\left[\begin{array}{cc} {\left({\hat{x}}_{c}^{\left(1\right)}(t|t)\right)}^{T} & {\left({\hat{x}}_{c}^{\left(2\right)}(t|t)\right)}^{T} \end{array}\right]}^{T},\;P_{c}^{\left(2\right)}(t|t)=\bar{N}\left[\begin{array}{cc} P_{c}^{\left(1\right)}(t|t) & P_{c}^{\left(12\right)}(t|t)\\ {\left(P_{c}^{\left(12\right)}(t|t)\right)}^{T} & P_{c}^{\left(2\right)}(t|t) \end{array}\right]{\bar{N}}^{T}.$$

**Proof.**  The proof is straightforward from ref. [24]. □

## 3. Main Results

**Figure 1:**
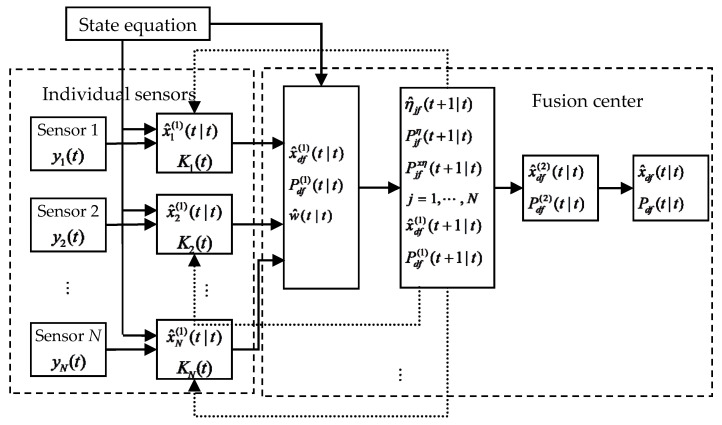
Globally optimal DFFWF.

In this section, we design the DFFWF ${\hat{x}}_{df}^{}(t|t)$ in Figure 1 based on local filter inputs ${\hat{x}}_{j}^{\left(1\right)}(t|t)$ and j=1,⋯,N. We first design the globally optimal DFFWF ${\hat{x}}_{df}^{\left(1\right)}(t|t)$ for the first ROS (8) using an innovation analysis approach. Then, the DFFWF ${\hat{x}}_{df}^{\left(2\right)}(t|t)$ for the second ROS can be obtained based on ${\hat{x}}_{df}^{\left(1\right)}(t|t)$ and the process noise filter $\hat{w}(t|t)$.

### 3.1. Local Filter with Feedback

In this subsection, we will derive the local filter ${\hat{x}}_{j}^{\left(1\right)}(t|t)$ based on the feedback information ${\hat{x}}_{df}^{\left(1\right)}(t|t-1)$, $P_{df}^{\left(1\right)}(t|t-1)$, ${\hat{\eta }}_{jf}^{}(t|t-1)=[0,I_{m_{j}},0]{\hat{\eta }}_{f}^{}(t|t-1)$, $P_{jf}^{\eta }(t|t-1)=[0,I_{m_{j}},0]P_{f}^{\eta }(t|t-1){\left[0,I_{m_{j}},0\right]}^{T}$ and $P_{jf}^{x\eta }(t|t-1)={\left[0,I_{m_{j}},0\right]}^{T}P_{f}^{x\eta }(t|t-1)$ from the fusion center to the individual sensor. ${\hat{x}}_{df}^{\left(1\right)}(t|t-1)$ and $P_{df}^{\left(1\right)}(t|t-1)$ are computed by Theorem 2, and ${\hat{\eta }}_{f}^{}(t|t-1)$, $P_{f}^{\eta }(t|t-1)$ and $P_{f}^{x\eta }(t|t-1)$ are computed by Theorem 3. In view of the definitions above, we know that ${\hat{\eta }}_{jf}^{}(t|t-1)$ is the *j*th row block of ${\hat{\eta }}_{f}^{}(t|t-1)$, and $P_{jf}^{\eta }(t|t-1)$ is the *j*th diagonal block of $P_{f}^{\eta }(t|t-1)$; $P_{jf}^{x\eta }(t|t-1)$ is the *j*th column block of $P_{f}^{x\eta }(t|t-1)$.

**Theorem** **1.** *For ROS (8) under Assumptions 1* *–5, the local state filter with feedback is given by:*

(28) $${\hat{x}}_{j}^{\left(1\right)}(t|t)={\hat{x}}_{df}^{\left(1\right)}(t|t-1)+K_{j}(t){\tilde{z}}_{j}(t),$$

(29) $$P_{j}^{\left(1\right)}(t|t)=P_{df}^{\left(1\right)}(t|t-1)-K_{j}(t)P_{j}^{z}(t)K_{j}^{T}(t).$$

*The gain matrix iscomputed by*

(30) $$K_{j}(t)=(P_{df}^{\left(1\right)}(t|t-1)D_{j}^{T}+P_{jf}^{x\eta }(t|t-1)){\left(P_{j}^{z}(t)\right)}^{-1},$$

*and innovation and its variance matrix are computedby*

(31) $${\tilde{z}}_{j}(t)=z_{j}(t)-D_{j}{\hat{x}}_{df}^{\left(1\right)}(t|t-1)-{\hat{\eta }}_{jf}^{}(t|t-1),$$

(32) $$P_{j}^{z}(t)=D_{j}P_{df}^{\left(1\right)}(t|t-1)D_{j}^{T}+P_{jf}^{\eta }(t|t-1)+D_{j}P_{jf}^{x\eta }(t|t-1)+{\left(P_{jf}^{x\eta }(t|t-1)\right)}^{T}D_{j}^{T},$$

*where fusion predictors*${\hat{x}}_{df}^{\left(1\right)}(t|t-1)$*and*${\hat{\eta }}_{jf}^{}(t|t-1)$*, covariance matrices*$P_{df}^{\left(1\right)}(t|t-1)$*and*$P_{jf}^{\eta }(t|t-1)$*and the cross-covariance matrix*$P_{jf}^{x\eta }(t|t-1)$*are the feedback information from the fusion center to the local filter. The initial values are*${\hat{x}}_{df}^{\left(1\right)}(0|-1)=x_{0}^{\left(1\right)}$*,*${\hat{\eta }}_{jf}^{}(0|-1)=0$*,*$P_{df}^{\left(1\right)}(0|-1)=P_{0}^{\left(1\right)}$*,*$P_{jf}^{\eta }(0|-1)=Q_{j}^{\eta }(0)$*and*$P_{jf}^{x\eta }(0|-1)=0$.

**Proof.**  Along the same line as the proof of CFF, the local filter can be obtained. The difference is that ${\hat{x}}_{df}^{\left(1\right)}(t|t-1)$, ${\hat{\eta }}_{jf}^{}(t|t-1)$, $P_{df}^{\left(1\right)}(t|t-1)$, $P_{jf}^{\eta }(t|t-1)$ and $P_{jf}^{x\eta }(t|t-1)$ are the feedback information, not the local information. □

### 3.2. Fusion Filter with Feedback

In the preceding subsection, we obtained the local filter based on the fusion state and measurement noise predictors. In this subsection, we will propose the fusion filter ${\hat{x}}_{df}^{\left(1\right)}(t|t)$ based on the local filter ${\hat{x}}_{j}^{\left(1\right)}(t|t)$ and its gain $K_{j}(t)$, j=1,⋯,N from individual sensors. In the fusion center, we regard local filters ${\hat{x}}_{j}^{\left(1\right)}(t|t)$ and j=1,⋯,N as measurement inputs. Let $\hat{x}(t|t)={\left[\begin{array}{ccc} {\left({\hat{x}}_{1}^{\left(1\right)}(t|t)\right)}^{T} & \cdots & {\left({\hat{x}}_{N}^{\left(1\right)}(t|t)\right)}^{T} \end{array}\right]}^{T}$, $K(t)=\mathrm{diag}(K_{1}(t),\cdots ,K_{N}(t))$. In the following text, we will derive the fusion filter ${\hat{x}}_{df}^{\left(1\right)}(t|t)$ based on the linear space $L(\hat{x}(0|0),\cdots ,\hat{x}(t|t))$ spanned by the measurement inputs ${\hat{x}}_{j}^{\left(1\right)}(t|t)$.

**Theorem** **2.** *For the first ROS (8) under Assumptions 1* *–5, in the fusion center, the DFFWF and its covariance matrix are provided by:*

(33) $${\hat{x}}_{df}^{\left(1\right)}(t|t)=(I_{n}-L(t)G){\hat{x}}_{df}^{\left(1\right)}(t|t-1)+L(t)\hat{x}(t|t),$$

(34) $$P_{df}^{\left(1\right)}(t|t)=P_{df}^{\left(1\right)}(t|t-1)-L(t)P^{\tilde{x}}(t)L^{T}(t),$$

*and the gain matrix is computed by*

(35) $$L(t)=(P_{df}^{\left(1\right)}(t|t-1)D^{T}+P_{f}^{x\eta }(t|t-1))K^{T}(t){\left(P^{\tilde{x}}(t)\right)}^{+},$$

*where*

(36) $$P^{\tilde{x}}(t)=K(t)(DP_{df}^{\left(1\right)}(t|t-1)D^{T}+P_{f}^{\eta }(t|t-1)+DP_{f}^{x\eta }(t|t-1)+{\left(DP_{f}^{x\eta }(t|t-1)\right)}^{T})K^{T}(t).$$

*The fusion predictor and its covariance matrix are computed by:*

(37) $${\hat{x}}_{df}^{\left(1\right)}(t+1|t)=\Phi {\hat{x}}_{df}^{\left(1\right)}(t|t)+\Gamma \hat{w}(t|t),$$

(38) $$P_{df}^{\left(1\right)}(t+1|t)=\Phi P_{df}^{\left(1\right)}(t|t)\Phi^{T}+\Gamma P^{w}(t|t)\Gamma^{T}+\Phi P^{xw}(t|t)\Gamma^{T}+{\left(\Phi P^{xw}(t|t)\Gamma^{T}\right)}^{T},$$

*where*$G={\left[I_{n},\cdots ,I_{n}\right]}^{T}$. $\hat{w}(t|t)$*,*$P^{w}(t|t)$*,*$P^{xw}(t|t)$*,*$P_{f}^{\eta }(t|t-1)$*and*$P_{f}^{x\eta }(t|t-1)$*are addressed in Theorem 3. The initial values are*${\hat{x}}_{df}^{\left(1\right)}(0|-1)=x_{0}^{\left(1\right)}$*,*$P_{df}^{\left(1\right)}(0|-1)=P_{0}^{\left(1\right)}$*,*$P_{f}^{\eta }(0|-1)=Q^{\eta }(0)$*and*$P_{f}^{x\eta }(0|-1)=0$.

**Proof.**  From the recursive projection formula [37], we obtain

(39) $${\hat{x}}_{df}^{\left(1\right)}(t|t)={\hat{x}}_{df}^{\left(1\right)}(t|t-1)+L(t)\tilde{x}(t),$$

where the innovation $\tilde{x}(t)$ and filtering gain matrix L(t) are defined as

(40) $$\tilde{x}(t)=\hat{x}(t|t)-\mathrm{proj}\text{\{}\hat{x}(t|t)|\hat{x}(0|0),\cdots ,\hat{x}(t-1|t-1)\},$$

(41) $$L(t)=E[x^{\left(1\right)}(t){\tilde{x}}^{T}(t)]{\left(P^{\tilde{x}}(t)\right)}^{+}.$$

From the local filter (28), the input to the fusion center $\hat{x}(t|t)$ can be expressed as

(42) $$\hat{x}(t|t)={\left[\begin{array}{ccc} {\left({\hat{x}}_{1}^{\left(1\right)}(t|t)\right)}^{T} & \cdots & {\left({\hat{x}}_{N}^{\left(1\right)}(t|t)\right)}^{T} \end{array}\right]}^{T}=G{\hat{x}}_{df}^{\left(1\right)}(t|t-1)+K(t){\left[\begin{array}{ccc} {\left({\tilde{z}}_{1}(t)\right)}^{T} & \cdots & {\left({\tilde{z}}_{N}(t)\right)}^{T} \end{array}\right]}^{T}.$$

Substituting the second equation of (8) and (31) into (42) and noting ${\tilde{\eta }}_{f}(t|t-1)={\left[\begin{array}{ccc} {\left({\tilde{\eta }}_{1f}(t|t-1)\right)}^{T} & \cdots & {\left({\tilde{\eta }}_{Nf}(t|t-1)\right)}^{T} \end{array}\right]}^{T}$, $\hat{x}(t|t)$ can be further rewritten as

(43) $$\hat{x}(t|t)=G{\hat{x}}_{df}^{\left(1\right)}(t|t-1)+K(t)(D{\tilde{x}}_{df}^{\left(1\right)}(t|t-1)+{\tilde{\eta }}_{f}(t|t-1)).$$

Applying ${\tilde{x}}_{df}^{\left(1\right)}(t|t-1)⊥L(\hat{x}(0|0),\cdots ,\hat{x}(t-1|t-1))$ and ${\tilde{\eta }}_{f}(t|t-1)⊥$$L(\hat{x}(0|0),\cdots ,\hat{x}(t-1|t-1))$, it follows that

(44) $$\mathrm{proj}\{\hat{x}(t|t)|\hat{x}(0|0),\cdots ,\hat{x}(t-1|t-1)\}=G{\hat{x}}_{df}^{\left(1\right)}(t|t-1),$$

which together with (39) and (40) yield (33). Using (43), the innovation associated with $\hat{x}(t|t)$ can be rewritten as

(45) $$\tilde{x}(t)=K(t)(D{\tilde{x}}_{df}^{\left(1\right)}(t|t-1)+{\tilde{\eta }}_{f}(t|t-1)).$$

Taking projection on both sides of the state update equation of (8) onto $L(\hat{x}(0|0),\cdots ,\hat{x}(t|t))$, (37) follows directly.

Subtracting (39) from $x^{\left(1\right)}(t)$ and (37) from $x^{\left(1\right)}(t+1)$, we obtain the filtering and prediction error equation for the state, respectively.

(46) $${\tilde{x}}_{df}^{\left(1\right)}(t|t)+L(t)\tilde{x}(t)={\tilde{x}}_{df}^{\left(1\right)}(t|t-1),$$

(47) $${\tilde{x}}_{df}^{\left(1\right)}(t+1|t)=\Phi {\tilde{x}}_{df}^{\left(1\right)}(t|t)+\Gamma \tilde{w}(t|t).$$

Noting that ${\tilde{x}}_{df}^{\left(1\right)}(t|t-1)⊥\tilde{x}(t)$ and substituting (46) and (47) into $P_{df}^{\left(1\right)}(t|t)=Ε[{\tilde{x}}_{df}^{\left(1\right)}(t|t){\left({\tilde{x}}_{df}^{\left(1\right)}(t|t)\right)}^{Τ}]$ and $P_{df}^{\left(1\right)}(t+1|t)=Ε[{\tilde{x}}_{df}^{\left(1\right)}(t+1|t){\left({\tilde{x}}_{df}^{\left(1\right)}(t+1|t)\right)}^{Τ}]$, (34) and (38) are obtained directly, where $P^{w}(t|t)=Ε[\tilde{w}(t|t){\tilde{w}}^{T}(t|t)]$ and $P^{xw}(t|t)=Ε[{\tilde{x}}_{df}^{\left(1\right)}(t|t){\tilde{w}}^{Τ}(t|t)]$. The proof is completed. □

**Theorem** **3.** *For the first ROS (8) under Assumptions 1* *–5, in the fusion center, the measurement noise predictor* ${\hat{\eta }}_{f}(t|t-1)$ *is computed by*

(48) $${\hat{\eta }}_{f}(t+1|t)=K^{\eta }(t+1|t)(\hat{x}(t|t)-G{\hat{x}}_{df}^{\left(1\right)}(t|t-1)),$$

(49) $$K^{\eta }(t+1|t)=Q^{\eta }(1)K^{T}(t){\left(P^{\tilde{x}}(t)\right)}^{+},$$

(50) $$P_{f}^{\eta }(t+1|t)=Q^{\eta }(0)-K^{\eta }(t+1|t)P^{\tilde{x}}(t){\left(K^{\eta }(t+1|t)\right)}^{T},$$

(51) $$P_{f}^{x\eta }(t+1|t)=\Gamma Q^{w\eta }(-1)-(\Phi L(t)+\Gamma K^{w}(t|t))P^{\tilde{x}}(t){\left(K^{\eta }(t+1|t)\right)}^{T}.$$

*The process noise filter is computed by*

(52) $$\hat{w}(t|t)=K^{w}(t|t)(\hat{x}(t|t)-G{\hat{x}}_{d}^{\left(1\right)}(t|t-1)),$$

(53) $$K^{w}(t|t)=Q^{w\eta }(0)K^{T}(t){\left(P^{\tilde{x}}(t)\right)}^{+},$$

(54) $$P^{w}(t|t)=Q^{w}-K^{w}(t|t)P^{\tilde{x}}(t){\left(K^{w}(t|t)\right)}^{T},$$

(55) $$P^{xw}(t|t)=-L(t)P^{\tilde{x}}(t){\left(K^{w}(t|t)\right)}^{T},$$

*where*$P^{\tilde{x}}(t)$*and*L(t)*are computed by Theorem 2. The initial value is* ${\hat{x}}_{df}^{\left(1\right)}(0|-1)=x_{0}^{\left(1\right)}$.

**Proof.**  From the recursive projection formula [37], it follows that

(56) $${\hat{\eta }}_{f}(t+1|t)={\hat{\eta }}_{f}(t+1|t-1)+K^{\eta }(t+1|t)\tilde{x}(t).$$

From the definition $\hat{x}(t|t)={\left[\begin{array}{ccc} {\left({\hat{x}}_{1}^{\left(1\right)}(t|t)\right)}^{T} & \cdots & {\left({\hat{x}}_{N}^{\left(1\right)}(t|t)\right)}^{T} \end{array}\right]}^{T}$, we obtain L(z(1),⋯,z(t−1)) $=L(\hat{x}(0|0),\cdots ,\hat{x}(t-1|t-1))$. According to Remark 3, we conclude that ${\hat{\eta }}_{f}(t+1|t-1)=0$. Substituting (40) and (44) into (56), we obtain measurement noise predictor (48), where the prediction gain matrix is defined by $K^{\eta }(t+1|t)=E[\eta (t+1){\tilde{x}}^{T}(t)]{\left(P^{\tilde{x}}(t)\right)}^{+}$. In view of (45), $\eta (t+1)⊥{\tilde{x}}_{df}^{\left(1\right)}(t|t-1)$ and $\eta (t+1)⊥L(\hat{x}(0|0),\cdots ,\hat{x}(t-1|t-1))$, it is known that

(57) $$E[\eta (t+1){\tilde{x}}^{T}(t)]=E[\eta (t+1)(D{\tilde{x}}_{df}^{\left(1\right)}(t|t-1)+{\tilde{\eta }}_{f}(t|t-1))]K^{T}(t)=E[\eta (t+1)\eta^{T}(t)]K^{T}(t),\;$$

which together with (10) yield the gain matrix (49). Subtracting (56) from η(t+1) and rearrangement, the error equation becomes ${\tilde{\eta }}_{f}(t+1|t)+K^{\eta }(t+1|t)\tilde{x}(t)=\eta (t+1)$, which together with ${\tilde{\eta }}_{f}(t+1|t)⊥\tilde{x}(t)$ yield (50). Similarly, the process noise filter (52) and filtering error variance (54) are obtained, where the gain matrix is defined by $K^{w}(t|t)=E[w(t){\tilde{x}}^{T}(t)]{\left(P^{\tilde{x}}(t)\right)}^{+}$.

Next, we derive $P_{f}^{x\eta }(t+1|t)=E[{\tilde{x}}_{df}^{\left(1\right)}(t+1|t){\tilde{\eta }}_{f}^{T}(t+1|t)]$. In view of ${\tilde{x}}_{df}^{\left(1\right)}(t+1|t)=x_{}^{\left(1\right)}(t+1)$ $-{\hat{x}}_{df}^{\left(1\right)}(t+1|t)$ and ${\hat{x}}_{df}^{\left(1\right)}(t+1|t)⊥{\tilde{\eta }}_{f}^{T}(t+1|t)$, the state update equation of (8) follows

(58) $$P_{f}^{x\eta }(t+1|t)=E[x_{}^{\left(1\right)}(t+1){\tilde{\eta }}_{f}^{T}(t+1|t)]=E[x_{}^{\left(1\right)}(t+1)\eta^{T}(t+1)]-E[x_{}^{\left(1\right)}(t+1){\tilde{x}}^{T}(t)]{\left(K^{\eta }(t+1|t)\right)}^{T}\;=\Gamma E[w(t)\eta^{T}(t+1)]-\{\Phi E[x_{}^{\left(1\right)}(t){\tilde{x}}^{T}(t)]+\Gamma E[w(t){\tilde{x}}^{T}(t)]\}{\left(K^{\eta }(t+1|t)\right)}^{T}.\;$$

According to (10) and the definition of L(t) and $K^{w}(t|t)$, we obtain $E[w(t)\eta^{T}(t+1)]=Q^{w\eta }(-1)$, $E[x_{}^{\left(1\right)}(t){\tilde{x}}^{T}(t)]=L(t)P^{\tilde{x}}(t)$ and $E[w(t){\tilde{x}}^{T}(t)]=K^{w}(t|t)P^{\tilde{x}}(t)$, which together with (58) yield (51). Similarly, we obtain (55). The proof is completed. □

**Remark** **4.** *It is worth noting that* $\hat{w}(t|t)$ *,* $K^{w}(t|t)$ *,* $P^{w}(t|t)$ *and* $P^{xw}(t|t)$ *computed in the fusion center are used to produce the fusion one-step predictor (37) and do not need to be sent to the local filter.*

**Corollary** **1.** *For the second ROS (6) under Assumptions 1* *–5, the DFFWF and its covariance matrix are computed by:*

(59) $${\hat{x}}_{df}^{\left(2\right)}(t|t)=B{\hat{x}}_{df}^{\left(1\right)}(t|t)+C\hat{w}(t|t),$$

(60) $$P_{df}^{\left(2\right)}(t|t)=BP_{df}^{\left(1\right)}(t|t)B^{T}+C\hat{w}(t|t)C^{T}+BP^{xw}(t|t)+{\left(BP^{xw}(t|t)\right)}^{T}.$$

*The cross-covariance matrix between the two subsystems is computed by*

(61) $$P_{df}^{\left(12\right)}(t|t)=P_{df}^{\left(1\right)}(t|t)B^{T}+P^{xw}(t|t)C^{T}.$$

*The fusion state filter and its filtering error covariance matrix of the original descriptor (1)–(3) are provided by*

(62) $${\hat{x}}_{df}^{}(t|t)=\bar{N}{\left[\begin{array}{cc} {\left({\hat{x}}_{df}^{\left(1\right)}(t|t)\right)}^{T} & {\left({\hat{x}}_{df}^{\left(2\right)}(t|t)\right)}^{T} \end{array}\right]}^{T},\;P_{df}^{}(t|t)=\bar{N}\left[\begin{array}{cc} P_{df}^{\left(1\right)}(t|t) & P_{df}^{\left(12\right)}(t|t)\\ {\left(P_{df}^{\left(12\right)}(t|t)\right)}^{T} & P_{df}^{\left(2\right)}(t|t) \end{array}\right]{\bar{N}}^{T}.$$

**Proof.**  The proof is straightforward from ref. [24]. □

To describe the implementation of the proposed DFFWF algorithm clearly and intuitively, the following Algorithm 1 environment is used:
**Algorithm 1**. The DFFWF Algorithm**Initialization**: Set the initial values ${\hat{x}}_{df}^{\left(1\right)}(0|-1)=x_{0}^{\left(1\right)}$, $P_{df}^{\left(1\right)}(0|-1)=P_{0}^{\left(1\right)}$, $P_{jf}^{\eta }(0|-1)=Q_{j}^{\eta }(0)$$\;\mathrm{and}\;P_{jf}^{x\eta }(0|-1)=0$ in each individual sensor and the initial values ${\hat{x}}_{df}^{\left(1\right)}(0|-1)=x_{0}^{\left(1\right)}$, $P_{df}^{\left(1\right)}(0|-1)=P_{0}^{\left(1\right)}$, $P_{f}^{\eta }(0|-1)=Q_{}^{\eta }(0)$$\;\mathrm{and}\;P_{f}^{x\eta }(0|-1)=0$ in the fusion center.**for t:=1 to N do** (if there are N samples)Step 1:Compute local filter ${\hat{x}}_{j}^{\left(1\right)}(t|t)$ and gains $K_{j}(t)$ based on Theorem 1 in each individual sensor.Step 2:Send ${\hat{x}}_{j}^{\left(1\right)}(t|t)$ and $K_{j}(t)$ to the fusion center.Step 3:Read all local filters ${\hat{x}}_{j}^{\left(1\right)}(t|t)$ and filter gains $K_{j}(t)$ to produce the augmented measurement input $\hat{x}(t|t)={\left[\begin{array}{ccc} {\left({\hat{x}}_{1}^{\left(1\right)}(t|t)\right)}^{T} & \cdots & {\left({\hat{x}}_{N}^{\left(1\right)}(t|t)\right)}^{T} \end{array}\right]}^{T}$ in the fusion center.Step 4:Compute DFFWF ${\hat{x}}_{df}^{\left(1\right)}(t|t)$ for the first ROS by (33)–(36) in Theorem 2.Step 5:Compute the measurement noise filter ${\hat{\eta }}_{f}(t+1|t)$ by (48)–(51) in Theorem 3.Step 6:Compute the process noise filter $\hat{w}(t|t)$ by (52)–(55) in Theorem 3.Step 7:Compute the fusion one-step predictor ${\hat{x}}_{df}^{\left(1\right)}(t+1|t)$ by (37) and its variance matrix $P_{df}^{\left(1\right)}(t+1|t)$ using (38).Step 8:Send ${\hat{x}}_{df}^{\left(1\right)}(t+1|t)$, $P_{df}^{\left(1\right)}(t+1|t)$, ${\hat{\eta }}_{jf}(t+1|t)=[0,I_{m_{j}},0]{\hat{\eta }}_{f}(t+1|t)$, $P_{jf}^{\eta }(t|t-1)=[0,I_{m_{j}},0]P_{f}^{\eta }(t|t-1){\left[0,I_{m_{j}},0\right]}^{T}$ $\mathrm{and}\;P_{jf}^{x\eta }(t|t-1)={\left[0,I_{m_{j}},0\right]}^{T}P_{f}^{x\eta }(t|t-1)$ to each individual sensor.Step 9:Compute DFFWF ${\hat{x}}_{df}^{\left(2\right)}(t|t)$ for the second ROS by (59) in Corollary 1.Step 10:Compute DFFWF ${\hat{x}}_{df}^{}(t|t)$ and its covariance matrix $P_{df}^{}(t|t)$ for the original descriptor by (61)–(62) in Corollary 1.
Output the DFFWF ${\hat{x}}_{df}^{}(t|t)$ and $P_{df}^{}(t|t)$. Step 11: **if t==N break** ***else*** set t=t+1, return to step 1. **end**

### 3.3. Estimation Performance of the DFFWF

In the proceeding subsection, we obtained the DFFWF ${\hat{x}}_{df}^{}(t|t)$ that has better reliability, flexibility and robustness since the used measurements in the fusion center are not raw measurements but the local filters ${\hat{x}}_{j}^{\left(1\right)}(t|t)$, j=1,⋯,N, that have been received from individual sensors. Subsequently, let us analyze the global optimality of the proposed DFFWF. According to Lemma 2 and Corollary 1, it is clear that the global optimality of ${\hat{x}}_{df}^{\left(1\right)}(t|t)$ implies the global optimality of ${\hat{x}}_{df}^{}(t|t)$. Without loss of generality, here we just analyze the global optimality of ${\hat{x}}_{df}^{\left(1\right)}(t|t)$.

**Lemma** **3.** *Let*A(t) *be a full column rank matrix and*R(t) *be a non-singular matrix. Then, we have*

$$A^{T}(t){\left[A(t)R(t)A^{T}(t)\right]}^{+}A(t)=R^{-1}(t).$$

**Theorem 4.**  *For the first ROS (8) under Assumptions 1* *–* *5* *, if* $K_{j}(t)$ *,* j=1,⋯,N *are of full column rank, the DFFWF is equivalent to the CFF, i.e., under the same initial values*

$${\hat{x}}_{df}^{\left(1\right)}(0|-1)={\hat{x}}_{c}^{\left(1\right)}(0|-1)=x_{0}^{\left(1\right)},\;{\hat{\eta }}_{c}^{}(0|-1)={\hat{\eta }}_{f}^{}(0|-1)=0,\;P_{df}^{\left(1\right)}(0|-1)=P_{c}^{\left(1\right)}(0|-1)=P_{0}^{\left(1\right)},\;P_{c}^{\eta }(0|-1)=P_{f}^{\eta }(0|-1)=Q^{\eta }(0),\;P_{c}^{x\eta }(0|-1)=P_{f}^{x\eta }(0|-1)=0.$$

*The following results hold:*

$${\hat{x}}_{df}^{\left(1\right)}(t|t)={\hat{x}}_{c}^{\left(1\right)}(t|t),P_{df}^{\left(1\right)}(t|t)=P_{c}^{\left(1\right)}(t|t),\forall \;t\geq 0.$$

**Proof**.  Substituting (43) into (33), the fusion filter becomes

(63) $${\hat{x}}_{df}^{\left(1\right)}(t|t)={\hat{x}}_{df}^{\left(1\right)}(t|t-1)+L(t)K(t)(D{\tilde{x}}_{df}^{\left(1\right)}(t|t-1)+{\tilde{\eta }}_{f}(t|t-1)).$$

Substituting (36) into (34), the fusion filtering error equation becomes

(64) $$P_{df}^{\left(1\right)}(t|t)=P_{df}^{\left(1\right)}(t|t-1)-L(t)K(t)[DP_{df}^{\left(1\right)}(t|t-1)D^{T}+P_{f}^{\eta }(t|t-1)\;+DP_{f}^{x\eta }(t|t-1)+{\left(DP_{f}^{x\eta }(t|t-1)\right)}^{T}]K^{T}(t)L^{T}(t).$$

It follows from (35), (49) and (53) that

(65) $$L(t)K(t)=(P_{df}^{\left(1\right)}(t|t-1)D^{T}+P_{f}^{x\eta }(t|t-1))K^{T}(t){\left(P^{\tilde{x}}(t)\right)}^{+}K(t),$$

(66) $$K^{\eta }(t+1|t)K(t)=Q^{\eta }(1)K^{T}(t){\left(P^{\tilde{x}}(t)\right)}^{+}K(t),$$

(67) $$K^{w}(t|t)K(t)=Q^{w\eta }(0)K^{T}(t){\left(P^{\tilde{x}}(t)\right)}^{+}K(t).$$

If $K_{j}(t)$, j=1,⋯,N are of full column rank, K(t) can be guaranteed to be of full column rank. By applying Lemma 3 and (24), we obtain

(68) $$K(t){\left(P^{\tilde{x}}(t)\right)}^{+}K^{T}(t)={\left(P_{c}^{z}(t)\right)}^{-1}.$$

Substituting (68) into (65)–(67) and comparing with (13), (17) and (53), we obtain

(69) $$L(t)K(t)=K_{c}(t),\;K^{\eta }(t+1|t)K(t)=K_{c}^{\eta }(t+1|t),\;K^{w}(t|t)K(t)=K_{c}^{w}(t|t),$$

which shows that L(t)K(t) and $K^{\eta }(t+1|t)K(t)$ are the centralized optimal estimation gain matrices $K_{c}(t)$ for state and $K_{c}^{\eta }(t+1|t)$ for measurement noise, respectively. Further we obtain $K_{c}(t)P_{c}^{z}(t)K_{c}^{T}(t)=L(t)P^{\tilde{x}}(t)L^{T}(t)$, which shows that $P_{df}^{\left(1\right)}(t|t)=P_{c}^{\left(1\right)}(t|t)$ for the same initial values $P_{df}^{\left(1\right)}(0|-1)=P_{c}^{\left(1\right)}(0|-1)=P_{0}^{\left(1\right)}$, $P_{c}^{\eta }(0|-1)=P_{f}^{\eta }(0|-1)=Q^{\eta }(0)$ and $P_{c}^{x\eta }(0|-1)=P_{f}^{x\eta }(0|-1)=0$. Similarly, we obtain $P_{df}^{\left(1\right)}(t+1|t)=P_{c}^{\left(1\right)}(t+1|t)$, $P_{c}^{\eta }(t+1|t)=P_{f}^{\eta }(t+1|t)$ and $P_{c}^{x\eta }(t+1|t)=P_{f}^{x\eta }(t+1|t)$.

Substituting (31) into (28) and noting the definition $\hat{x}(t|t)={\left[{\left({\hat{x}}_{1}^{\left(1\right)}(t|t)\right)}^{T}\cdots {\left({\hat{x}}_{N}^{\left(1\right)}(t|t)\right)}^{T}\right]}^{T}$, $z(t)={\left[z_{1}^{T}(t),\cdots ,z_{N}^{T}(t)\right]}^{T}$ and ${\hat{\eta }}_{c}(t|t-1)={\left[{\left({\hat{\eta }}_{1}^{}(t|t-1)\right)}^{T}\cdots {\left({\hat{\eta }}_{N}^{}(t|t-1)\right)}^{T}\right]}^{T}$, $\hat{x}(t|t)$ can be expressed as

(70) $$\hat{x}(t|t)=(G-K(t)D){\hat{x}}_{df}^{\left(1\right)}(t|t-1)+K(t)z(t)-K(t){\hat{\eta }}_{c}(t|t-1).$$

Substituting (70) into (33) and comparing with (11), we obtain ${\hat{x}}_{df}^{\left(1\right)}(t|t)={\hat{x}}_{c}^{\left(1\right)}(t|t)$ under the same initial values ${\hat{x}}_{df}^{\left(1\right)}(0|-1)={\hat{x}}_{c}^{\left(1\right)}(0|-1)=x_{0}^{\left(1\right)}$ and ${\hat{\eta }}_{c}^{}(0|-1)={\hat{\eta }}_{f}^{}(0|-1)=0$. Similarly, we obtain ${\hat{x}}_{c}^{\left(1\right)}(t+1|t)={\hat{x}}_{df}^{\left(1\right)}(t+1|t)$ and ${\hat{\eta }}_{c}^{}(t+1|t)={\hat{\eta }}_{f}^{}(t+1|t)$. The proof is completed. □

**Remark 5**.  *In Theorem 4, the global optimality of DFFWF algorithm is analyzed. Now, we compare the computational cost with distributed fusion filter weighted by matrices (DFFWM). Here, we give the computational cost by calculating the times of multiplication and division. For ease of comparisons, without loss of generality, we only give the computational cost of the first ROS. In the fusion center, DFFWF and DFFWM have the same computational cost, the computational order of magnitude is* $O\left({\left(Nn_{1}\right)}^{3}\right)$ *. Hence, the proposed DFFWF algorithm is superior to the DFFWM in accuracy, which will be shown in the simulation research.*

## 4. Simulation Research

In this section, we use a numerical example and a circuit system to illustrate the estimation performance of the proposed fusion filtering algorithm.

**Example 1.**  *Consider a numerical example described in ref. [7]:*

(71) $$\left[\begin{array}{cccc} -2.13 & 0 & 0 & 0\\ 1 & 0.5 & 0 & 0\\ 1 & 0.5 & 0 & 0\\ 0 & -1 & 0 & 0 \end{array}\right]x(t+1)=\left[\begin{array}{cccc} -1 & 0.2 & 0 & 0\\ -0.5 & 0 & 0 & 0\\ 1 & -0.5 & -0.5 & 0\\ 0 & -1 & -1 & 2 \end{array}\right]x(t)+\left[\begin{array}{cc} 0.5 & 0\\ 0 & 0.8\\ 0.8 & 0\\ 0 & -0.6 \end{array}\right]w(t),$$

(72) $$y_{j}(t)={\bar{D}}_{j}x(t)+v_{j}(t),$$

(73) $$v_{j}(t+1)={\bar{U}}_{j}v_{j}(t)+\mu_{j}(t),j=1,2,3,$$

*where*w(t)*and*$\mu_{j}(t)$*,*j=1,2,3*are mutually uncorrelated zero mean white noises with variances*$Q^{w}$ *and*$Q_{j}^{\mu }$*. We know from (71) that*$\bar{M}=\bar{N}=I_{4}$*since the original descriptor is already the canonical form. In the simulation, we set*${\bar{D}}_{1}=\left[\begin{array}{cccc} 1 & 0.5 & 1 & 0\\ 0 & 1 & 0 & 1 \end{array}\right]$*,*${\bar{D}}_{2}=\left[\begin{array}{cccc} 1 & 0 & 0 & 1\\ 0 & 0.8 & 1 & 0 \end{array}\right]$*,*${\bar{D}}_{3}=\left[\begin{array}{cccc} 0.1 & 0.6 & 1 & 1\\ 1 & 0.1 & 0 & 1 \end{array}\right]$*,*${\bar{U}}_{1}=0.2$*,*${\bar{U}}_{2}=0.5$*,*${\bar{U}}_{3}=0.3$*,*$Q^{w}=I_{2}$*,*$Q_{1}^{\mu }=2I_{2}$*,*$Q_{2}^{\mu }=3I_{2}$*,*$Q_{3}^{\mu }=I_{2}$*and*$x(0)={\left[0,0,0,0\right]}^{T}$*,*$v_{j}(0)={\left[0,0\right]}^{T}$*,*j=1,2,3*. To analyze theglobal optimality, wetake the initial values*${\hat{x}}_{df}^{\left(1\right)}(0|-1)={\hat{x}}_{c}^{\left(1\right)}(0|-1)={\left[0,0\right]}^{T}$*,*$P_{0}^{\left(1\right)}=0.1I_{2}$*,* $P_{0}^{v_{j}}=0.1I_{2}$*and*j=1,2,3.

**Figure 2:**
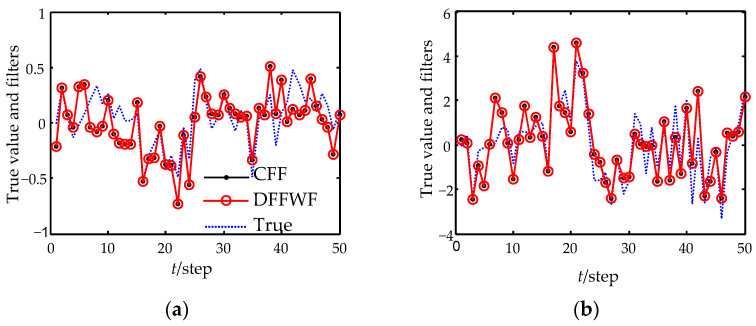

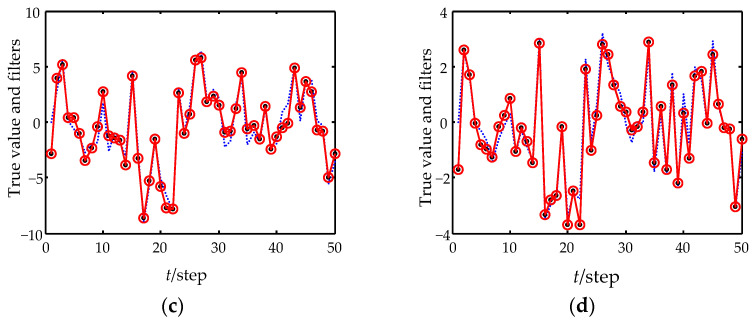
Tracking performance of DFFWF and CFF. (**a**) The first state component. (**b**) The second state component. (**c**) The third state component. (**d**) The fourth state component.

**Figure 3:**
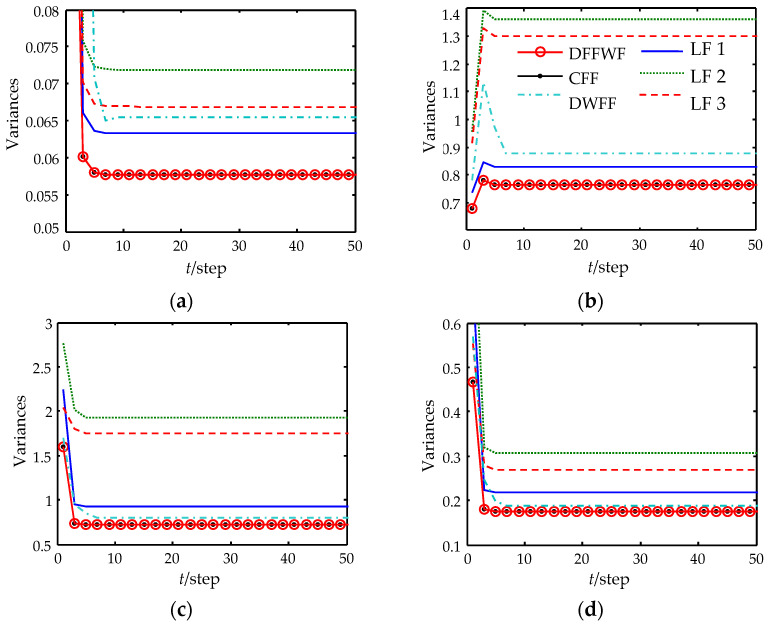
Filtering error variances of DFFWF, CFF and all local filters. (**a**) The first state component. (**b**) The second state component. (**c**) The third state component. (**d**) The fourth state component.

Figure 2 shows the expected tracking performances of the proposed DFFWF and the CFF. Figure 3 shows the filtering error variances of DFFWF, CFF, DFFWF and all local filters with feedback (LFWF). In Figure 2 and Figure 3, each curve is drawn at each 2-step. The true values and filters are given in Table 1 at time 0 and 50. From Figure 2 and Figure 3 and Table 1, it is conclude that the designed DFFWF is numerically equivalent to the CFF for the same initial values. That is to say the designed DFFWF also has global optimality. To show the superiority of the proposed DFFWF, DFFWM is also computed and shown in Figure 3. It shows that estimation accuracy of the proposed DFFWF is higher than that of any LFWF and DFFWM. Moreover, for the first and second components, the estimation accuracy of DFFWM is lower than that of LFWF measured by sensor 1. But for the third and fourth components, the result is just the opposite. The reason is that DFFWM is obtained by weighting all the local filters without feedback. On the other hand, DFFWF requires the feedback communication from the fusion center to individual sensors.

**Figure 4:**
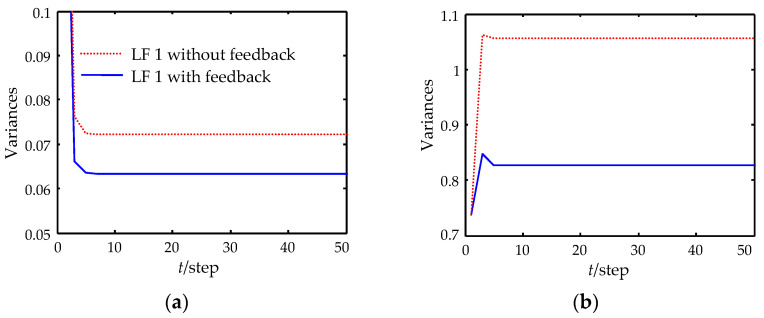

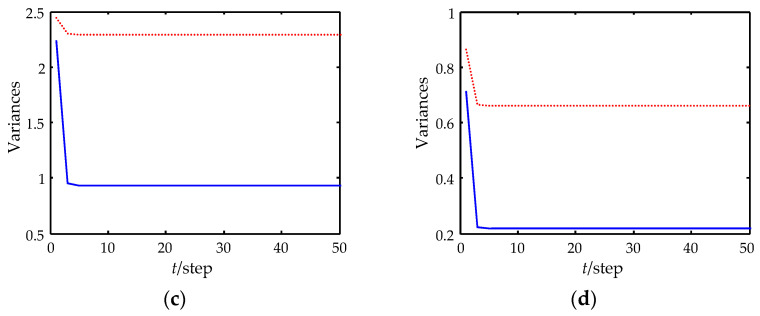
Filtering error variances of local filters with and without feedback for sensor 1. (**a**) The first state component. (**b**) The second state component. (**c**) The third state component. (**d**) The fourth state component.

Figure 4 shows the filtering error variances of LFWF with and without feedback for sensor 1. It is clear that the estimation accuracy of the proposed local filter with feedback is higher than that of the local filter without feedback, which demonstrates that feedback does improve the local estimation accuracy.

**Figure 5:**
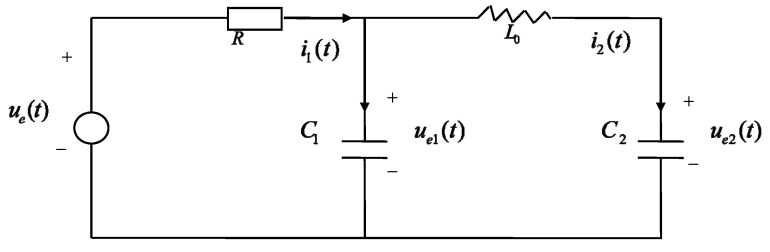
The circuit system.

**Example 2.**  *Consider the circuit system measured by three sensors shown in Figure 5, where the voltage source*$u_{e}$*is the control input. It is effected by white noise*w(t)*due to the equipment installation, circuitry interference and voltage fluctuation. For*R, $L_{0}$ *and*$C_{i}$, i=1,2 *denote the resistor, inductor and the ith capacity, respectively. Selecting the state*$x(t)={\left[u_{e1}(t),u_{e2}(t),i_{1}(t),i_{2}(t)\right]}^{T}$, $u_{e1}(t)$ *and*$i_{1}(t)$ *are the voltage and currents of*$C_{1}$, *and*$u_{e2}(t)$ *and*$i_{2}(t)$ *are the voltage and current of*$C_{2}$. *According to Kirchoff’s second law, we can establish the following state equation [1,11]:*

(74) $$\left[\begin{array}{cccc} C_{1} & 0 & 0 & 0\\ 0 & C_{2} & 0 & 0\\ 0 & 0 & {-L}_{0} & 0\\ 0 & 0 & 0 & 0 \end{array}\right]\dot{x}(t)=\left[\begin{array}{cccc} 0 & 0 & 0 & 1\\ 0 & 0 & 1 & 0\\ -1 & 1 & 0 & 0\\ 1 & 0 & R & R \end{array}\right]x(t)+\left[\begin{array}{c} 0\\ 0\\ 0\\ -1 \end{array}\right]u_{e}(t)+\left[\begin{array}{c} 0\\ 0\\ 0\\ -1 \end{array}\right]w(t).$$

*The measurement equation is the same as in example 1. Taking the sample period* $T_{0}=0.05\ll 1$ *from Euler’s approximation, the corresponding discrete-time model can be obtained as:*

(75) $$\left[\begin{array}{cccc} C_{1} & 0 & 0 & 0\\ 0 & C_{2} & 0 & 0\\ 0 & 0 & -L_{0} & 0\\ 0 & 0 & 0 & 0 \end{array}\right]x(t+1)=\left[\begin{array}{cccc} C_{1} & 0 & 0 & T_{0}\\ 0 & C_{2} & T_{0} & 0\\ -T_{0} & T_{0} & -L_{0} & 0\\ T_{0} & 0 & T_{0}R & T_{0}R \end{array}\right]x(t)+\left[\begin{array}{c} 0\\ 0\\ 0\\ -T_{0} \end{array}\right]u_{e}(t)+\left[\begin{array}{c} 0\\ 0\\ 0\\ -T_{0} \end{array}\right]w(t).$$

**Figure 6:**
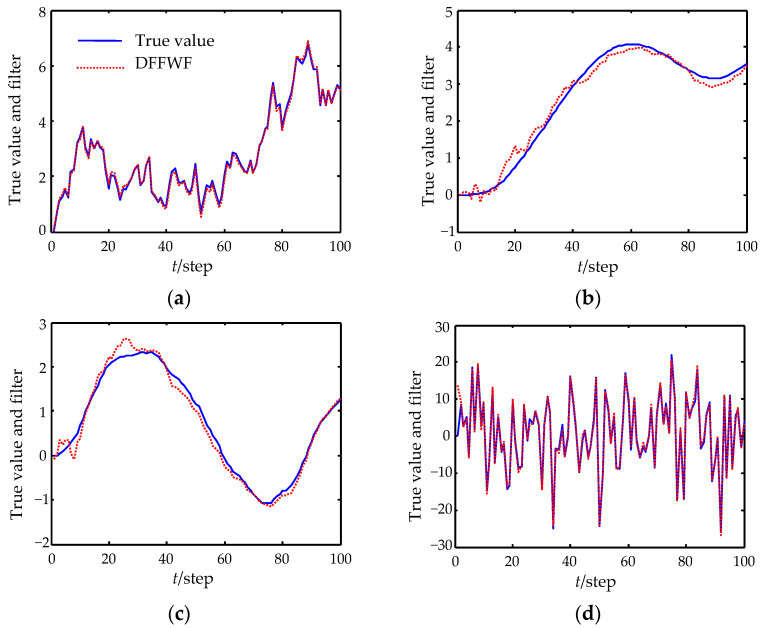
Tracking performance of DFFWF. (**a**) The voltage of $C_{1}$. (**b**) The voltage of $C_{2}$. (**c**) The current of $C_{1}$. (**d**) The current of $C_{2}$.

In the simulation, we set $C_{1}=1$, $C_{2}=1$, $L_{0}=1$, R=2, $u_{e}=5$, $H_{1}=[1,0,0,1]$, $H_{2}=[0,0,1,0]$, $H_{3}=[0,1,0,0.5]$, $Q^{w}=1$, $Q_{1}^{\mu }=0.5$, $Q_{2}^{\mu }=2$, $Q_{3}^{\mu }=1$, ${\bar{U}}_{1}=0.2$, ${\bar{U}}_{2}=0.5$, ${\bar{U}}_{3}=0.3$. Select $\bar{M}={\bar{B}}^{-1}$, $\bar{N}=I_{4}$, then the parameters in (4) can be obtained as ${\bar{A}}_{1}=\left[\begin{array}{ccc} 1.0230 & 0.0026 & 0.0510\\ 0.0026 & 0.9975 & -0.0497\\ -0.0510 & 0.0497 & 0.9950 \end{array}\right]$, ${\bar{A}}_{2}=[-0.4605,-0.0510,-1.0205]$, ${\bar{B}}_{1}=I_{3}$, ${\bar{B}}_{2}=[0,0,0]$ and ${\bar{B}}_{3}=1$. The filtering performance is provided in Figure 6. It shows the expected tracking results.

## 5. Conclusions

This paper investigated the problem of distributed fusion filters was investigated for multi-sensor descriptor systems with time-correlated measurement noise. Using singular value decomposition, the original descriptor system was transformed into two reduced-order non-descriptor subsystems. First, an equivalent new system with a new measurement noise was established using a different approach to remove the time-correlated measurement noises. The new measurement noise was one-step auto- and cross-correlated. Based on the local measurement and fusion predictor from the fusion center, the local filters were obtained in the LMV sense. Then, the local filters and filtering gains were sent to the fusion center and used as the measurement inputs to produce the fusion filters. Under the condition that all local filtering gains were of full column rank, the presented DFFWF has global optimality. Furthermore, the obtained feedback can also improve the estimation of each local filter. In the future, we will try to deal with the state estimation problem for descriptor systems with time-correlated noises and some network-induced phenomena such as random transmission delays [12,38], losses [36,39] and deception attacks [40].

## Figures and Tables

**Table 1:** True values and filters of SFF and CFF.

Sample	State	True Value	DFFWF	CFF
0	$x_{1}^{\left(1\right)}(t)$	0	−0.2175	−0.2175
	$x_{2}^{\left(1\right)}(t)$	0	0.2240	0.2240
	$x_{1}^{\left(2\right)}(t)$	0	−2.8612	−2.8612
	$x_{2}^{\left(2\right)}(t)$	0	−1.6882	−1.6882
50	$x_{1}^{\left(1\right)}(t)$	0.1383	0.0710	0.0710
	$x_{2}^{\left(1\right)}(t)$	2.5727	2.1459	2.1459
	$x_{1}^{\left(2\right)}(t)$	−3.5400	−2.8476	−2.8476
	$x_{2}^{\left(2\right)}(t)$	−1.0143	−0.6017	−0.6017

## Data Availability

Not applicable.
